# Human C-terminal *CUBN*﻿﻿ variants associate with chronic proteinuria and normal renal function

**DOI:** 10.1172/JCI161852

**Published:** 2022-06-01

**Authors:** Mathilda Bedin, Olivia Boyer, Aude Servais, Yong Li, Laure Villoing-Gaudé, Marie-Josephe Tête, Alexandra Cambier, Julien Hogan, Veronique Baudouin, Saoussen Krid, Albert Bensman, Florie Lammens, Ferielle Louillet, Bruno Ranchin, Cecile Vigneau, Iseline Bouteau, Corinne Isnard-Bagnis, Christoph J. Mache, Tobias Schäfer, Lars Pape, Markus Gödel, Tobias B. Huber, Marcus Benz, Günter Klaus, Matthias Hansen, Kay Latta, Olivier Gribouval, Vincent Morinière, Carole Tournant, Maik Grohmann, Elisa Kuhn, Timo Wagner, Christine Bole-Feysot, Fabienne Jabot-Hanin, Patrick Nitschké, Tarunveer S. Ahluwalia, Anna Köttgen, Christian Brix Folsted Andersen, Carsten Bergmann, Corinne Antignac, Matias Simons

Original citation: *J Clin Invest*. 2020;130(1):335–344. https://doi.org/10.1172/JCI129937

Citation for this corrigendum: *J Clin Invest*. 2022;132(11):e161852. https://doi.org/10.1172/JCI161852

The authors recently became aware that the standard errors for the diabetes group were incorrect in Supplemental Table 9. The supplemental data PDF has been updated with the correct information. This correction affected the *P* values, but not the effect sizes and directionalities. The authors have stated that all conclusions of the paper remain the same.

The authors regret the errors.

## Version 1 06/01/2022

Electronic publication

